# Simultaneous cannabis and psychedelic use among festival and concert attendees in Colorado: characterizing enhancement and adverse reactions using mixed methods

**DOI:** 10.1186/s42238-024-00235-x

**Published:** 2024-07-11

**Authors:** Cianna J. Piercey, Eleftherios Hetelekides, Hollis C. Karoly

**Affiliations:** Department of Psychology, Colorado State University, Fort Collins, CO 80521 USA

**Keywords:** Polysubstance use, Cannabis, Psychedelics, Concerts, Music festivals, Thematic analysis

## Abstract

**Background:**

Most studies examining the simultaneous use of cannabis with other drugs have focused on cannabis and alcohol, with fewer studies examining simultaneous use of cannabis with other drugs. The United States is currently experiencing an upward trend in psychedelic use and there is an increasing need to characterize cannabis and psychedelic drug interactions to best inform public health recommendations.

**Materials and Methods:**

A mixed methods field study design was used to survey participants (N = 128) on their lifetime co-use of cannabis with other drugs. Participants who reported lifetime co-use of cannabis and psychedelics (N = 63) were then asked open-ended questions about their most recent simultaneous co-use experience (i.e., how cannabis enhanced their psychedelic experience and whether they experienced any adverse reactions). We conducted a thematic analysis of responses describing how cannabis enhanced the psychedelic experience (N = 54). However, due to low response rate for participants reporting an adverse reaction (N = 7, 11.1%), responses to this question were not analyzed thematically and are instead presented individually.

**Results:**

Themes included tension reduction and balancing of drug effects (N = 27, 50%), enhancement to psychological processes (N = 11, 20.4%), intensified psychedelic drug effects (N = 12, 22.2%), enhanced psychedelic come-down experience (N = 8, 14.8%), and overall ambiguous enhancement (N = 7, 13%). Among participants reporting an adverse reaction, individual responses included increased anxiety and intensity of the experience, decreased sociability, increased negative affect, sleepiness, disassociation, and confusion.

**Conclusion:**

Additional research is warranted to better characterize cannabis and psychedelic drug interactions to best inform public health recommendations.

**Supplementary Information:**

The online version contains supplementary material available at 10.1186/s42238-024-00235-x.

## Introduction

Polysubstance use, including simultaneous (i.e., at the same time such that effects overlap) and concurrent use (i.e., within a timeframe such as past year or month), is common among people who use cannabis (Davis et al. 2019; Pape et al. 2009; Crummy et al. 2020). For example, recent national findings suggest that over 90% of past 30-day cannabis users also used other substances in the past month (Carlini and Schauer 2022). To date, most studies examining the simultaneous use of cannabis with other substances have focused on cannabis and alcohol (Waddell et al. 2023; Stevens et al. 2021; Karoly et al. 2022), with fewer studies examining simultaneous use of cannabis with other drugs. Several states and cities in the U.S. have recently decriminalized psychedelic substances (e.g., N,N-Dimethyltryptamine [DMT], psilocybin) with further reform efforts underway across the country. Additionally, an estimated 8.5 million Americans used psychedelics in 2022 (Center for Behavioral Health Statistics S 2022) and this number may be expected to increase in response to a changing legal and social landscape.

Cannabis is commonly used during recreational psychedelic experiences (Grov et al. 2009; Licht et al. 2022) and evidence suggests that combined use may be associated with a more intense psychedelic experience. For example, Kuc and colleagues (2022) found a linear positive relationship between cannabis dose (operationalized as none, low, medium, or high dose) and subjective effects of psychedelics (i.e., mystical-type experiences, ego-dissolution and visual alterations) in an online survey of participants’ most recent psychedelic experience. A quadratic relationship between cannabis dose and challenging experiences was also found, such that low doses were associated with lower scores on the Challenging Experiences Questionnaire (CEQ) (Barrett et al. 2016), while high doses were associated with higher CEQ scores. In addition to intensifying the psychedelic experience, cannabis has been found to be used in combination with drugs such as 3,4-Methyl​enedioxy​methamphetamine (MDMA) and psilocybin to enhance the psychedelic experience and reduce negative effects associated with certain drugs (Hunt et al. 2009).

Prior research has linked co-use of cannabis and psychedelics (e.g., Lysergic acid diethylamide [LSD], MDMA, ketamine) with adverse effects (Palamar et al. 2016). Further, evidence from the Global Drug Survey indicates that among individuals seeking emergency medical treatment for psilocybin and LSD exposure in 2017, cannabis was potentially implicated in 37% of psilocybin-related and 50% of LSD-related cases (Kopra et al. 2022a & b). Additionally, in a large international survey of challenging psilocybin experiences, 53% of participants reported using cannabis during or prior to the challenging experience (Carbonaro et al. 2016). Interestingly, however, cannabis use was found to be inversely associated with overall subjective difficulty of the experience and 25% of participants endorsed using cannabis to alleviate symptoms of the challenging experience. Moreover, among participants who reported using cannabis to mitigate the challenging experience, 50% reported that using cannabis helped them substantially.

These findings underscore a complex interplay between the effects of cannabis and psychedelics on acute subjective experiences that warrants further exploration. In the current study, we sought to investigate the nuances described above and further characterize interactions between cannabis and psychedelic compounds through a mixed methods field survey of simultaneous cannabis and psychedelic co-use. A mixed methods approach was selected to elevate the voices of individuals with experience using this drug combination and participants were recruited from a context in which cannabis and psychedelic use is common (music festivals and concerts) (Palamar and Keyes 2020). Specifically, participants were asked to describe the ways cannabis enhanced their most recent psychedelic experience and report on any negative or adverse reactions they may have experienced. To further characterize cannabis consumption patterns within the context of psychedelic experiences, participants were also asked to report information related to cannabis product type (e.g., flower, concentrates, edibles), quantity, potency, and frequency with which cannabis was used during the psychedelic experience.

## Method

### Participants

Participants (Table 1) were 128 concert and music festival attendees and were included in the present analysis if they endorsed having ever used cannabis and psychedelics simultaneously (*N* = 63). Participants were excluded from the study if they exhibited visible signs of intoxication at the time of recruitment or were not between the ages of 18–65. The study was approved by Colorado State University’s Institutional Review Board.

**Table 1:** Respondent characteristics

Characteristics	N	%	M	SD	Min	Max
Age			28.48	5.72	18	46
Gender						
Agender	2	3.2				
Gender fluid	1	1.6				
Man	26	41.3				
Woman	26	41.3				
Non-binary	1	1.6				
Prefer not to answer	2	3.2				
Transgender						
Yes	1	1.6				
No	58	92.1				
Prefer not to answer	1	1.6				
Ethnicity						
Arab, Middle Eastern, or North African	4	6.3				
Asian or Asian American	4	6.3				
Black or African American	2	3.2				
Hispanic or Latino	12	19.0				
Native American or Alaska Native	2	3.2				
Native Hawaiian or Other Pacific Islander	1	1.6				
White or European American	31	49.2				
Not listed	1	1.6				
Prefer not to answer	3	4.8				
Race						
Asian	2	3.2				
Black	1	1.6				
Indigenous, Aboriginal, or First Nations	1	1.6				
Hispanic or Latino	12	19.0				
Middle Eastern	1	1.6				
White	40	63.5				
Sexual Orientation						
Straight or heterosexual	38	60.3				
Gay	2	3.2				
Bisexual	12	19.0				
Pansexual	5	7.9				
Sexually fluid	2	3.2				
Queer	1	1.6				
Demisexual	2	3.2				
Questioning	1	1.6				
I use a different term	1	1.6				
Prefer not to answer	2	3.2				
Cannabis use frequency						
No current use or less than once per year	5	8.0				
Less than once per month	4	6.4				
Monthly use	2	3.2				
Weekly use	13	20.6				
Daily use	35	55.6				

*M* mean, *SD* standard deviation

### Procedure

Concert and festival attendees were approached and asked to complete a 15-min field survey on their substance use while tailgating, standing in line waiting to enter an event, or from festival campgrounds. Data collection took place in Colorado and included events such as Sonic Bloom Festival, Global Dance Festival, and concerts at Red Rocks Amphitheater. Attendees who agreed to take part in the study scanned a QR code linking them to the survey or were provided with an iPad to complete the survey if their personal device was uncharged or did not have service. Following completion of the survey, participants were immediately compensated with a commemorative art print created by the first author of the study. Free harm reduction supplies (e.g., fentanyl test strips, naloxone) were offered to all individuals approached by researchers, regardless of their decision to participate.

### Measures

#### Substance use

Participants were asked to report whether they have ever used cannabis at the same time as another substance and selected from a list of 22 drugs which substances they have simultaneously co-used with cannabis in their lifetime. If participants endorsed a prior experience of simultaneously co-using cannabis with at least one psychedelic substance, they were asked to report on their most recent co-use experience. Specifically, participants were provided with the following prompt at the beginning of this question set: “The following questions will ask you to describe your most recent experience using cannabis at the same time as a psychedelic substance.” Participants selected from a list which psychedelic substance(s) they used and what form(s) of cannabis they used (i.e., flower, concentrates, or edibles). Participants were also asked to report the quantity, potency (Cuttler et al. 2017), and within-session frequency (i.e., the number of times they used cannabis during their psychedelic experience) of the cannabis product they used. Participants also reported on their typical frequency of cannabis use. Descriptive statistics for co-use variables are reported in Table 2, with outlying values windzorized (Tabachnick and Fidell 2012).

**Table 2:** Characteristics of the products used during participants’ most recent co-use experience

Characteristics	N	%	M	SD	Min	Max
Psychedelic(s) used						
LSD	26	41.3				
Psilocybin	32	50.8				
DMT	3	4.8				
Mescaline	2	3.2				
MDMA	17	27.0				
Ketamine	11	17.5				
Cannabis product(s) used						
Flower	50	79.4				
Concentrates	32	50.8				
Edibles	14	22.2				
Flower characteristics						
Quantity (grams)			3.76	3.89	0.2	14
Within-session frequency (times used)			6.02	5.95	1	21
THC content						
0–4%	2	4.0				
5–9%	1	2.0				
10–14%	1	2.0				
15–19%	7	14.0				
20–24%	12	24.0				
25–30%	9	18.0				
Greater than 30%	3	6.0				
Unsure	15	30.0				
Concentrate Characteristics						
Dab quantity (# of dabs)			4.06	4.67	1	21
Cartridge quantity (# of hits)			11.21	7.90	2	21
Dab within-session frequency (times used)			3.67	2.94	1	11
Cartridge within-session frequency (times used)			8.67	6.89	1	21
Dab THC content						
Less than 50%	3	15.0				
50–59%	1	5.0				
60–69%	1	5.0				
70–79%	8	40.0				
80–90%	1	5.0				
Greater than 90%	0	0.0				
Unsure	6	30.0				
Cartridge THC content						
Less than 50%	4	18.2				
50–59%	2	9.1				
60–69%	2	9.1				
70–79%	2	9.1				
80–90%	7	31.8				
Greater than 90%	0	0.0				
Unsure	5	22.7				
Edible Characteristics						
Edibles within-session frequency (times used			1.73	1.39	1	6
Edibles THC content (milligrams)			70.00	69.85	5	200

#### Open-ended survey questions

Participants were asked the following open-ended questions 1) “In what ways did using cannabis with the psychedelic enhance the experience?” and 2) “Did you experience any negative or adverse reactions due to using cannabis with the psychedelic?”.

### Data analysis

We conducted a thematic analysis of responses to the open-ended survey item “In what ways did using cannabis with the psychedelic enhance the experience?” Specifically, we familiarized ourselves with participant responses, generated initial codes, searched for themes, and reviewed and refined themes until 100% agreement was reached between coders (Braun and Clarke 2006). Because we aimed to address a specific research question, we only coded data that was relevant to the specific question asked (Maguire et al. 2017). Additionally, due to the exploratory nature of the study and paucity of prior research in this area, we used an open-coding system (i.e., codes were developed and modified while working through the coding process) rather than pre-defined codes (Maguire et al. 2017). Responses to the second open-ended question (related to adverse and negative reactions) were not thematically analyzed due to low response rate, and are instead individually reported.

## Results

Among participants who endorsed co-use of cannabis and psychedelics (*N* = 63), 50.8% reported using cannabis most recently with psilocybin, 41.3% reported using with LSD, 27% reported using with MDMA, 17.5% reported using with ketamine, 4.8% reported using with DMT, and 3.2% reported using with mescaline. When asked what forms of cannabis participants used during their most recent co-use experience, 79.4% of participants reported using flower cannabis, 50.8% reported using concentrates, and 22.2% of participants reported using edibles. Notably, 33.3% of participants endorsed using more than one psychedelic substance during their most recent co-use experience and 39.7% of participants reported using more than one form of cannabis. Further, most participants reporting on their most recent co-use experience endorsed daily (55.6%) or weekly (20.6%) cannabis use.

For flower products, the mean quantity used was 3.76 g (SD = 3.89), the mean within-session frequency of use (number of times used during the experience) was 6.02 (SD = 5.95), and the most commonly endorsed THC content was 20–24%. For dab products (e.g., shatter, wax), the mean number of dabs used per session was 4.06 (SD = 4.67), the mean within-session frequency of use was 3.67 (SD = 2.94), and the most commonly endorsed THC content was 70–79%. For concentrate cartridge products, the mean number of hits taken was 11.21 (SD = 7.90), the mean within-session frequency was 8.67 (SD = 6.89), and the most commonly endorsed THC content was 80–90%. Finally, for edible products, the mean within-session frequency was 1.73 (SD = 1.39) and the mean THC content was 70 mg (SD = 69.85). Descriptive statistics for the cannabis and psychedelic products that participants reported using is reported in Table 2. In the supplementary material (supplement 1–6), descriptive statistics for these variables are also provided across each individual theme and for participants reporting an adverse reaction. Across the 63 participants who reported on their most recent co-use experience, 54 provided a written response detailing the ways that cannabis enhanced their most recent psychedelic experience. Our final codebook included 13 codes, of which 5 themes were generated from the data (Table 3).

**Table 3:** Themes related to enhancement

Main Theme	Subthemes	Example Participant Responses
Tension reduction/ balancing of psychedelic drug effects (*N* = 27)	N/A	“Helps ease my anxiety and mellows me out” “Cannabis helps calm me down, and during a bad trip especially” “Balanced me out”
Enhancement to psychological processes (*N* = 11)	Ambiguous enhancement to psychological processes (*N* = 2) Increased positive affect (*N* = 6) Cognitive enhancement (*N* = 5)	“Weed makes everything better and more manageable” “Made me feel so chill and happy” “Just enhanced the joy” “Changed mental state to a much more introspective state” “Helped to make me feel less out of control”
Intensified psychedelic drug effects (*N* = 12)	Ambiguous enhancement of drug effects (*N* = 3) Enhanced visual effects (*N* = 7) Enhanced body effects (*N* = 4)	“Sometimes makes me trip harder” “It intensified the visuals” “It brought me more into my body”
Enhanced psychedelic come-down (*N* = 8)	Extension of psychedelic drug effects/intensified come-down (*N* = 7) Enhanced recovery from psychedelic drug effects (*N* = 2)	“It helped to re-invigorate my trip in the latter 3 h of my psychedelic experience” “I always use after, while coming down. Helps to prolong or wind down”
Overall ambiguous enhancement i.e., participant reported enhancement but did not elaborate (*N* = 7)	N/A	“Absolutely enhanced it” “Made everything better” “Enhanced”

### Theme 1: tension reduction and balancing of psychedelic drug effects

The most commonly endorsed theme was tension reduction and balancing of psychedelic drug effects (N = 27, 50%). Specifically, participants reported using cannabis to mitigate anxiety related to the psychedelic experience or to calm them down during a “bad trip”. Some participants also shared that using cannabis allowed them to “stay grounded” and others stated that cannabis served to balance psychedelic drug effects. Among participants endorsing theme 1, 24 participants (88.9%) reported using flower, 12 participants (44.4%) reported using concentrates, and 3 participants (11.1%) reported using edibles. Across psychedelic substances, 12 participants reported using LSD (44.4%), 13 reported using psilocybin (48.1%), 1 reported using DMT (3.7%), 9 reported using MDMA (33.3%), and 7 reported using ketamine (25.9%).

### Theme 2: enhancement to psychological processes

Participants reported that using cannabis during the psychedelic experience enhanced psychological processes within three distinct domains (*N* = 11, 20.4%), which we have denoted as subthemes. Subthemes included cognitive enhancement (*N* = 5, 9.3%), increased positive affect (*N* = 6, 11.1%), and ambiguous enhancement to psychological processes (*N* = 2, 3.7%). Among participants endorsing theme 2, 10 participants (90.9%) reported using flower, 6 participants (54.5%) reported using concentrates, and 2 participants (18.2%) reported using edibles. Across psychedelic substances, 6 participants reported using LSD (54.5%), 6 reported using psilocybin (54.5%), 1 reported using DMT (9.1%), 3 reported using MDMA (27.3%), and 1 reported using ketamine (9.1%).

#### Subtheme 2.1: cognitive enhancement

Participants shared that cannabis enhanced cognitive experiences while using psychedelics by allowing for greater cognitive control and attenuation of intrusive thoughts. Additionally, some participants reported that using cannabis led to a “much more introspective state” and allowed them to “think more clearly and honestly” while using psychedelics.

#### Subtheme 2.2: increased positive affect

Participants reported increased feelings of joy and happiness while co-using cannabis with psychedelics and noted that using cannabis made the psychedelic experience more enjoyable.

#### Subtheme 2.3: ambiguous psychological enhancement

Responses were categorized as ambiguous psychological enhancement when participants reported psychological enhancement but did not specify in enough detail to be coded as cognitive or emotional enhancement (e.g., “weed makes everything better and more manageable”).

### Theme 3: intensified psychedelic drug effects

Participants reported that using cannabis during their most recent psychedelic experience intensified psychedelic drug effects (*N* = 12, 22.2%), again encompassing three domains. Subthemes included enhanced visual effects (*N* = 7, 13%), enhanced body effects (*N* = 4, 7.4%), and ambiguous enhancement of drug effects (*N* = 3, 5.6%). Among participants endorsing theme 3, 11 participants (91.7%) reported using flower, 7 participants (58.3%) reported using concentrates, and 4 participants (33.3%) reported using edibles. Across psychedelic substances, 5 participants reported using LSD (41.7%), 7 reported using psilocybin (58.3%), 1 reported using mescaline (8.3%), 3 reported using MDMA (25%), and 3 reported using ketamine (25%).

#### Subtheme 3.1: enhanced visual effects

Participants reported that using cannabis made visual effects feel stronger. While most participants did not specify beyond increased intensity of visual effects, one participant noted enhancement of colors and lights.

#### Subtheme 3.2: enhanced body effects

Participants shared that cannabis “improved the bodily feeling” when using psychedelics, enhanced the “body high”, and brought them “more into their body”.

#### Subtheme 3.3: ambiguous enhancement of drug effects

Responses were categorized as ambiguous enhancement of drug effects when participants reported that their drug experience felt more intense but did not specify in what ways (e.g., “makes me trip harder”).

### Theme 4: enhanced psychedelic “come-down” experience

Participants reported that using cannabis enhanced their psychedelic “come-down” (*N* = 8, 14.8%) through the following mechanisms: 1) extending the psychedelic experience or intensifying the come-down period (*N* = 7, 13%) and 2) aiding in recovery from psychedelic drug effects (*N* = 2, 3.7%). Among participants endorsing theme 4, 3 participants (37.5%) reported using flower, 4 participants (50%) reported using concentrates, and 2 participants (25%) reported using edibles. Across psychedelic substances, 3 participants reported using LSD (37.5%), 5 reported using psilocybin (62.5%), 1 reported using DMT (12.5%), 1 reported using mescaline (12.5%), and 1 reported using MDMA (12.5%).

#### Subtheme 4.1: extension of psychedelic drug effects or intensified come-down

Participants shared that using cannabis made their psychedelic experience last longer, “brought back the trip”, and made the latter hours of the trip feel more intense.

#### Subtheme 4.2: enhanced recovery from psychedelic drug effects

Several participants reported enhanced recovery from psychedelic drug effects such that using cannabis helped participants fall asleep after the psychedelic experience or “wind down”.

### Theme 5: overall ambiguous enhancement

Finally, several participants (*N* = 7, 13%) reported that cannabis enhanced their psychedelic experience but did not specify the mechanism by which it was enhanced (e.g., “made everything better”). Among participants endorsing theme 5, 4 participants (57.1%) reported using flower, 6 participants (85.7%) reported using concentrates, and 2 participants (28.6%) reported using edibles. Across psychedelic substances, 4 participants reported using LSD (57.1%), 3 reported using psilocybin (42.9%), 1 reported using DMT (14.3%), 4 reported using MDMA (57.1%), and 1 reported using ketamine (14.3%).

### Negative or adverse reactions

11.1% of participants (*N* = 7) responded to the question inquiring about negative or adverse reactions. Though not thematically analyzed due to low response rate, responses to this question included content around increased anxiety and intensity of the experience, decreased sociability, increased negative affect, sleepiness, disassociation, and confusion (these responses are listed in Table 4). Among participants reporting an adverse reaction, 5 participants (71.4%) reported using flower, 3 participants (42.9%) reported using concentrates, and 1 participant (14.3%) reported using edibles. Across psychedelic substances, 3 participants reported using LSD (42.9%), 3 reported using psilocybin (42.9%), 1 reported using DMT (14.3%), 2 reported using MDMA (28.6%), and 1 reported using ketamine (14.3%).

**Table 4:** Adverse reactions reported by participants

Individual Participant Responses
“Maybe sleepy / drowsy”
“Makes me more confused and less social”
“It’s hard to tell if the unwanted dmt was all I was experiencing but I was in a dissociative nightmare of a trip”
“Dissociated a bit, cried”
“Brought on higher level of anxiety, uncomfortably intense”
“Possible intensity”
“Increased waves of anxiety”

## Discussion

We sought to explore qualitatively how cannabis might enhance the psychedelic experience or contribute to negative or adverse experiences in a non-clinical sample of festival and concert attendees in Colorado. Results corroborate prior studies showing that cannabis may intensify the psychedelic experience (Kuc et al. 2022). Additionally, our study extends a growing body of evidence that some individuals report using cannabis to mitigate challenging psychedelic experiences and specifically acute anxious symptoms (Carbonaro et al. 2016). These qualitative themes support what is currently known about the pharmacological effects of cannabis and possible interactions with psychedelic substances. The primary chemical constituents of cannabis that are responsible for the psychoactive effects of the plant (Δ-9 tetrahydrocannabinol, THC, and cannabidiol, CBD) are known to exert their effects mainly through cannabinoid receptors (CB1 and CB2) (An et al. 2020). However, there is also evidence that CBD can act as an inverse agonist on human serotonin (5-HT) receptors (Martínez-Aguirre et al. 2020) and chronic exposure to THC may promote hallucinogenic-related signaling via 5-HT_2A_ receptors in mice, though this hasn’t been tested in humans (Ibarra-Lecue et al. 2018).

Given shared actions of cannabinoid and psychedelic drugs on serotonin and other receptor targets in the brain (Halberstadt and Geyer 2011), as well as the commonly reported concomitant use of cannabis and psychedelics (Grov et al. 2009; Licht et al. 2022), it is crucial to clarify if and how interactions between the use of psychedelics and cannabis may promote positive experiences and exacerbate negative ones. For example, are frequent cannabis users less likely to experience negative/adverse reactions to simultaneous use compared to infrequent cannabis users? Are concentrated cannabis products more strongly associated with negative/adverse reactions compared to flower products? While lack of statistical power in the current study precludes quantitative analysis of whether individuals who reported a negative co-use experience differed on variables like cannabis use frequency, potency, and product type used (Piercey et al. 2023), these are important empirical questions that may inform if/how individuals might use cannabis to promote positive experiences and ameliorate challenging ones. To inform larger scale future studies which may be better poised to answer these questions, we point readers to the supplementary material, where we report cannabis and psychedelic product characteristics across themes and among participants reporting an adverse reaction. However, our initial observations suggest that product characteristics among individuals reporting an adverse reaction were similar to that of the overall sample, and in some cases, amount of cannabis used and within-session frequency of use was lower.

Our finding that cannabis can prolong the psychedelic drug experience and intensify the “come down” period has important harm reduction and public health implications. Understanding the course and duration of a drug’s effects is critical to engagement with risk management practices (e.g., arranging for a “trip sitter”) and planning for challenging experiences (Palmer and Maynard 2022). This finding also provides preliminary support for using cannabis as a substitute for “redosing” psychedelics. For example, an individual looking to “bring back the trip” may use cannabis to achieve their desired drug effect without needing to take more of the psychedelic. Likewise, co-using cannabis with psychedelics in recreational settings may allow individuals to achieve similar levels of intoxication while consuming a smaller initial psychedelic dose.

### Limitations and future directions

Limitations of the current study include its cross-sectional design and demographic homogeneity (i.e., most participants self-identified as White, cisgender, and heterosexual). Additionally, most participants reporting on their most recent co-use experience endorsed daily or weekly cannabis use, which may have impacted findings due to factors such as tolerance or level of experience with cannabis. The study also relied on a sample of festival and concert attendees in Colorado (i.e., results may not be generalizable to other populations) and did not collect information related to frequency of psychedelic use. Further, the wording of our open-ended enhancement question encouraged participants to discuss enhancement specifically, which may have limited participants’ discussion of other subjective effects or use motives. Likewise, we did not provide a definition of negative or adverse effects in our open-ended adverse effects question, which may have caused participants to interpret these terms differently. We do note however, that in asking the question in this way, we sought to implement a person-centered approach and avoid imposing a definition of a “negative experience” onto participants.

In addition to addressing the limitations described above, future research should better characterize adverse reactions, including how factors like cannabis use frequency, tolerance, and product type may promote positive experiences and/or exacerbate risks. Future studies should also collect information pertaining to psychedelic dose and order of co-use (e.g., cannabis before psychedelics or psychedelics before cannabis), and examine differences in subjective effects with regard to specific drug combinations (e.g., cannabis and psilocybin vs. cannabis and LSD).

### Supplementary Information

Supplementary Material 1.

## Data Availability

Data and materials available from the corresponding author upon reasonable request.

## Abbreviations

DMT: N,N-Dimethyltryptamine
CEQ: Challenging Experiences Questionnaire
THC: Δ-9 Tetrahydrocannabinol
CBD: Cannabidiol
CB1: Cannabinoid receptor 1
CB2: Cannabinoid receptor 2
5-HT: 5-Hydroxytryptamine
LSD: Lysergic acid diethylamide
MDMA: 3,4-Methyl enedioxy methamphetamine
