# Supplementary material for: Simultaneous cannabis and psychedelic use among festival and concert attendees in Colorado: characterizing enhancement and adverse reactions using mixed methods
Source: J Cannabis Res. 2024 Jul 11;6:29. doi: 10.1186/s42238-024-00235-x (PMC11238454; doi:10.1186/s42238-024-00235-x)
Supplement: Supplementary file 1 — Supplementary Material 1. [file 42238_2024_235_MOESM1_ESM.pdf]

Supplement 1. Cannabis and Psychedelic Product Descriptives for Theme 1: Tension Reduction and Balancing of Psychedelic Drug Effects

Characteristics	N	%	M	SD	Min	Max
Psychedelic(s) used						
LSD	12	44.4				
Psilocybin	13	48.1				
DMT	1	3.7				
Mescaline	0	0				
MDMA	9	33.3				
Ketamine	7	25.9				
Cannabis product(s) used						
Flower	24	88.9				
Concentrates	12	44.4				
Edibles	3	11.1				
Flower characteristics						
Quantity (grams)			4.81	4.00	0.50	14.00
Within-session frequency (times used)			6.27	5.49	1.00	21.00
THC content						
0-4%	1	3.7				
5-9%	1	3.7				
10-14%	1	3.7				
15-19%	5	18.5				
20-24%	6	22.2				
25-30%	3	11.1				
Greater than 30%	2	7.4				
Unsure	5	18.5				
Missing	3	11.1				
Concentrate Characteristics						
Dab quantity (# of dabs)			3.40	2.41	1.00	6.00
Cartridge quantity (# of hits)			13.86	9.03	2.00	21.00
Dab within-session frequency (times used)			5.80	4.44	1.00	11.00
Cartridge within-session frequency (times used)			10.83	9.26	1.00	21.00
Dab THC content						
Less than 60%	0	0				
60-69%	1	3.7				
70-79%	4	14.8				
80-90%	0	0				
Greater than 90%	0	0				
Unsure	2	7.4				
Missing	20	74.1				
Cartridge THC content						
Less than 50%	1	3.7				
50-59%	1	3.7				
60-69%	1	3.7				
70-79%	0	0				
80-90%	2	7.4				
Greater than 90%	0	0				
Unsure	3	11.1				
Missing	19	70.4				
Edible Characteristics						
Edibles THC content (milligrams)			70.00	51.96	10.00	100.00
Edibles within-session frequency (times used)			1.20	0.45	1.00	2.00

Supplement 2. Cannabis and Psychedelic Product Descriptives for Theme 2: Enhancement to Psychological Processes

Characteristics	N	%	M	SD	Min	Max
Psychedelic(s) used						
LSD	6	54.5				
Psilocybin	6	54.5				
DMT	1	9.1				
Mescaline	0	0				
MDMA	3	27.3				
Ketamine	1	9.1				
Cannabis product(s) used						
Flower	10	90.9				
Concentrates	6	54.5				
Edibles	2	18.2				
Flower characteristics						
Quantity (grams)			4.78	5.27	0.20	14.00
Within-session frequency (times used)			5.78	6.26	1.00	21.00
THC content						
0-4%	0	0				
5-9%	0	0				
10-14%	0	0				
15-19%	2	18.2				
20-24%	3	27.3				
25-30%	1	9.1				
Greater than 30%	1	9.1				
Unsure	3	27.3				
Missing	1	9.1				
Concentrate Characteristics						
Dab quantity (# of dabs)			8.25	8.62	3.00	21.00
Cartridge quantity (# of hits)			11.33	9.07	3.00	21.00
Dab within-session frequency (times used)			3.33	0.58	3.00	4.00
Cartridge within-session frequency (times used)			7.50	3.54	5.00	10.00
Dab THC content						
Less than 50%	0	0				
50-59%	1	9.1				
60-69%	0	0				
70-79%	1	9.1				
80-90%	1	9.1				
Greater than 90%	0	0				
Unsure	2	18.2				
Missing	6	54.5				
Cartridge THC content						
Less than 50%	1	9.1				
50-59%	0	0				
60-69%	2	18.2				
70-79%	0	0				
80-90%	0	0				
Greater than 90%	0	0				
Unsure	1	9.1				
Missing	7	63.6				
Edible Characteristics						
Edibles THC content (milligrams)			65.00	49.50	30.00	100.00
Edibles within-session frequency (times used)			2.00	1.00	1.00	3.00

Supplement 3. Cannabis and Psychedelic Product Descriptives for Theme 3: Intensified Psychedelic Drug Effects

Characteristics	N	%	M	SD	Min	Max
Psychedelic(s) used						
LSD	5	41.7				
Psilocybin	7	58.3				
DMT	0	0				
Mescaline	1	8.3				
MDMA	3	25.0				
Ketamine	3	25.0				
Cannabis product(s) used						
Flower	11	91.7				
Concentrates	7	58.3				
Edibles	4	33.3				
Flower characteristics						
Quantity (grams)			2.36	2.56	0.25	8.00
Within-session frequency (times used)			4.70	3.59	1.00	12.00
THC content						
0-4%	0	0				
5-9%	0	0				
10-14%	1	8.3				
15-19%	2	16.7				
20-24%	4	33.3				
25-30%	2	16.7				
Greater than 30%	0	0				
Unsure	2	16.7				
Missing	1	8.3				
Concentrate Characteristics						
Dab quantity (# of dabs)			2.75	0.96	2.00	4.00
Cartridge quantity (# of hits)			13.00	7.53	4.00	21.00
Dab within-session frequency (times used)			5.25	3.86	3.00	11.00
Cartridge within-session frequency (times used)			11.25	7.09	4.00	21.00
Dab THC content						
Less than 50%	1	8.3				
50-59%	0	0				
60-69%	1	8.3				
70-79%	1	8.3				
80-90%	1	8.3				
Greater than 90%	0	0				
Unsure	0	0				
Missing	8	66.7				
Cartridge THC content						
Less than 50%	1	8.3				
50-59%	1	8.3				
60-69%	0	0				
70-79%	0	0				
80-90%	2	16.7				
Greater than 90%	0	0				
Unsure	0	0				
Missing	8	66.7				
Edible Characteristics						
Edibles THC content (milligrams)			78.75	91.87	5.00	200.00
Edibles within-session frequency (times used)			1.25	0.50	1.00	2.00

Supplement 4. Cannabis and Psychedelic Product Descriptives for Theme 4: Enhanced Psychedelic "Come-down" Experience

Characteristics	N	%	M	SD	Min	Max
Psychedelic(s) used						
LSD	3	37.5				
Psilocybin	5	62.5				
DMT	1	12.5				
Mescaline	1	12.5				
MDMA	1	12.5				
Ketamine	0	0				
Cannabis product(s) used						
Flower	3	37.5				
Concentrates	4	50.0				
Edibles	2	25.0				
Flower characteristics						
Quantity (grams)			3.50	NA	3.50	3.50
Within-session frequency (times used)			12.67	8.02	5.00	21.00
THC content						
0-4%	0	0				
5-9%	0	0				
10-14%	0	0				
15-19%	1	12.5				
20-24%	1	12.5				
25-30%	0	0				
Greater than 30%	0	0				
Unsure	1	12.5				
Missing	5	62.5				
Concentrate Characteristics						
Dab quantity (# of dabs)			2.50	0.71	2.00	3.00
Cartridge quantity (# of hits)			15.50	7.78	10.00	21.00
Dab within-session frequency (times used)			2.00	0.00	2.00	2.00
Cartridge within-session frequency (times used)			15.50	7.78	10.00	21.00
Dab THC content						
Less than 50%	0	0				
50-59%	0	0				
60-69%	0	0				
70-79%	1	12.5				
80-90%	0	0				
Greater than 90%	0	0				
Unsure	1	12.5				
Missing	6	75.0				
Cartridge THC content						
Less than 50%	0	0				
50-59%	0	0				
60-69%	0	0				
70-79%	0	0				
80-90%	2	25.0				
Greater than 90%	0	0				
Unsure	0	0				
Missing	6	75.0				
Edible Characteristics						
Edibles THC content (milligrams)			15.00	7.07	10.00	20.00
Edibles within-session frequency (times used)			1.00	NA	1.00	1.00

Supplement 5. Cannabis and Psychedelic Product Descriptives for Theme 5: Overall Ambiguous Enhancement

Characteristics	N	%	M	SD	Min	Max
Psychedelic(s) used						
LSD	4	57.1				
Psilocybin	3	42.9				
DMT	1	14.3				
Mescaline	0	0				
MDMA	4	57.1				
Ketamine	1	14.3				
Cannabis product(s) used						
Flower	4	57.1				
Concentrates	6	85.7				
Edibles	2	28.6				
Flower characteristics						
Quantity (grams)			5.10	6.93	0.20	10.00
Within-session frequency (times used)			16.33	8.08	7.00	21.00
THC content						
0-4%	0	0				
5-9%	0	0				
10-14%	0	0				
15-19%	0	0				
20-24%	0	0				
25-30%	1	14.3				
Greater than 30%	1	14.3				
Unsure	2	28.6				
Missing	3	42.9				
Concentrate Characteristics						
Dab quantity (# of dabs)			8.00	8.83	2.00	21.00
Cartridge quantity (# of hits)			9.33	10.12	3.00	21.00
Dab within-session frequency (times used)			3.67	0.58	3.00	4.00
Cartridge within-session frequency (times used)			4.50	0.71	4.00	5.00
Dab THC content						
Less than 50%	0	0				
50-59%	1	14.3				
60-69%	0	0				
70-79%	2	28.6				
80-90%	0	0				
Greater than 90%	0	0				
Unsure	2	28.6				
Missing	2	28.6				
Cartridge THC content						
Less than 50%	0	0				
50-59%	1	14.3				
60-69%	2	28.6				
70-79%	0	0				
80-90%	1	14.3				
Greater than 90%	0	0				
Unsure	0	0				
Missing	3	42.9				
Edible Characteristics						
Edibles THC content (milligrams)			65.00	49.50	30.00	100.00
Edibles within-session frequency (times used)			1.75	0.96	1.00	3.00

Supplement 6. Cannabis and Psychedelic Product Descriptives for Participants Reporting an Adverse Reaction

Characteristics	N	%	M	SD	Min	Max
Psychedelic(s) used						
LSD	3	42.9				
Psilocybin	3	42.9				
DMT	1	14.3				
Mescaline	0	0				
MDMA	2	28.6				
Ketamine	1	14.3				
Cannabis product(s) used						
Flower	5	71.4				
Concentrates	3	42.9				
Edibles	1	14.3				
Flower characteristics						
Quantity (grams)			3.00	5.04	0.25	12.00
Within-session frequency (times used)			3.00	1.58	1.00	5.00
THC content						
0-4%	0	0				
5-9%	0	0				
10-14%	0	0				
15-19%	2	28.6				
20-24%	0	0				
25-30%	0	0				
Greater than 30%	0	0				
Unsure	3	42.9				
Missing	2	28.6				
Concentrate Characteristics						
Dab quantity (# of dabs)			1.50	0.71	1.00	2.00
Cartridge quantity (# of hits)			4.50	3.54	2.00	7.00
Dab within-session frequency (times used)			1.50	0.71	1.00	2.00
Cartridge within-session frequency (times used)			2.00	1.41	1.00	3.00
Dab THC content						
Less than 50%	0	0				
50-59%	0	0				
60-69%	0	0				
70-79%	1	14.3				
80-90%	0	0				
Greater than 90%	0	0				
Unsure	1	14.3				
Missing	5	71.4				
Cartridge THC content						
Less than 50%	1	14.3				
50-59%	0	0				
60-69%	0	0				
70-79%	0	0				
80-90%	1	14.3				
Greater than 90%	0	0				
Unsure	0	0				
Missing	5	71.4				
Edible Characteristics						
Edibles THC content (milligrams)			5.00	N/A	5.00	5.00
Edibles within-session frequency (times used)			1.00	N/A	1.00	1.00
